# How should we interpret estimates of individual repeatability?

**DOI:** 10.1002/evl3.40

**Published:** 2018-01-31

**Authors:** Alastair J. Wilson

**Affiliations:** ^1^ Centre for Ecology and Conservation University of Exeter (Penryn Campus) Cornwall TR10 9FE United Kingdom

**Keywords:** Behaviour, Behavioural genetics, Phenotypic plasticity, Quantitative genetics

## Abstract

Individual repeatability (*R*), defined as the proportion of observed variance attributable to among‐individual differences, is a widely used summary statistic in evolutionarily motivated studies of morphology, life history, physiology and, especially, behaviour. Although statistical methods to estimate *R* are well known and widely available, there is a growing tendency for researchers to interpret *R* in ways that are subtly, but importantly, different. Some view *R* as a property of a dataset and a statistic to be interpreted agnostically with respect to mechanism. Others wish to isolate the contributions of ‘intrinsic’ and/or ‘permanent’ individual differences, and draw a distinction between true (intrinsic) and pseudo‐repeatability arising from uncontrolled extrinsic effects. This latter view proposes a narrower, more mechanistic interpretation, than the traditional concept of repeatability, but perhaps one that allows stronger evolutionary inference as a consequence (provided analytical pitfalls are successfully avoided). Neither perspective is incorrect, but if we are to avoid confusion and fruitless debate, there is a need for researchers to recognise this dichotomy, and to ensure clarity in relation to how, and why, a particular estimate of *R* is appropriate in any case.

Over recent years there has been an explosion of interest in evolutionary and behavioural ecology in characterising the extent to which individuals within populations show repeatable phenotypic differences across observations separated in time. From an evolutionary perspective, it is often noted that individual repeatability *R*, the proportion of variance attributable to among‐individual differences sets an upper bound for heritability (Falconer and Mackay 1996); but see (Dohm 2002) for exceptions). Similarly, natural selection occurs when trait differences among‐individuals cause fitness variation. Thus, trait variation among individuals is a necessary, but not sufficient, condition for genetic variation and selection, the two ingredients of adaptive phenotypic evolution. As a consequence, estimates of *R* have long been used to draw tentative evolutionary conclusions when formal quantitative genetic analyses are not possible (e.g., Bakker 1999; Conradsen et al. 2016). For instance, in studies of wild bird populations repeatabilities of reproductive traits (e.g., clutch size, lay date) have been estimated as proxies for heritability (e.g., Perrins and Jones 1974; Erikstad et al. 1993) and/or to separate the influences of territory and parental quality (Przybylo et al. 2001). For morphological traits, repeatabilities have been used to test predictions arising from handicap models of sexual selection. For example, Foley et al. (2012) argued that, for antler traits in cervids, *R* declines as conditions become more variable. Though not surprising in itself, this is consistent with the prediction that male traits under sexual selection should be honest signals as a consequence of condition dependence.

Although individual repeatabilities are estimated for all types of phenotypes, the recent surge of interest has focused particularly on behavioural traits (Bell et al. 2009). In this context, consistent differences among individuals are viewed as evidence of animal personality, coping style or behavioural syndromes. While preferred terminology varies among researchers (and according to whether the phenomenon is univariate or multivariate), animal personality is usually defined as the presence of behavioural differences among‐individuals that are repeatable across time and/or context. This definition has led empiricists to design studies that target multiple behavioural observations per individual subject, allowing statistical separation of among‐individual from within‐individual variance. The former provides the statistical signature of personality, with its effect size often standardised to a repeatability. This approach, though not without its critics in behavioural ecology (Beekman and Jordan 2017) has highlighted the fundamental evolutionary importance of (behavioural) variation at multiple hierarchical levels (e.g., within‐ and among‐individuals; Dingemanse 2017). This in turn has greatly facilitated inclusion of behavioural traits in integrative studies of multivariate phenotypes including, for instance, stress physiology (Boulton et al. 2015) and metabolism (Nespolo and Franco 2007; Auer et al. 2016).

However, while estimating R from appropriate data is straightforward using widely available, well‐documented methods (e.g., ANOVA, simple correlation analysis, linear‐mixed effect models; see e.g., Wolak et al. 2011), there are also growing differences among researchers in their biological interpretations of this parameter. For some it is a statistical parameter to be estimated and interpreted agnostically with respect to biological mechanisms driving variance. For others estimation of R is intended, either explicitly or implicitly, to capture specific drivers of among‐individual variance while excluding others. This note does not advocate a single ‘correct’ approach. Indeed my primary aim is to highlight that different, but equally correct views coexist in the literature that differ in their biological interpretation. Nonetheless, some incorrect—or at least misleading–‐practices also persist. A secondary aim here is to demonstrate one such practice (whereby scaling traits to ratios can make interpretation of repeatability estimates problematic) and to present its simple solution. Thereby I hope to (i) help researchers draw appropriate understanding from published results, and (ii) encourage authors to be more explicit in what they want *R* to represent and why.

## Divergent Concepts of Individual Repeatability

Repeatability is a very widely used statistical concept and thus is not, in any general sense, a parameter with specific evolutionary, behavioural, or indeed biological meaning. *R* simply describes the proportion of variance in a measured quantity that is attributable to some factor or class by which observations can be grouped. It is thus a standardised measure of among‐class variance. Equivalently, it describes the extent of correlation among observations within levels of the grouping factor, hence its alternative moniker as the intraclass correlation (ICC). In organismal biology the grouping factor of interest is commonly the identity of an individual animal such that *R* = V_I_/V_P_ (where, for some trait of interest, V_I_is the among‐individual variance and V_P_ is the total phenotypic variance). Thus, in this context, *R* tells us the extent to which observations on the same individuals are correlated and provides an intuitive measure of variation among‐individuals.

Two points follow from the above. First, while being a widely used measure of among individual variance, *R* is the ratio of V_I_ to V_P_ and the latter includes both within‐ and among‐individual components. Thus *R* is an outcome of the variance partitioned in a particular dataset, and is dependent on among‐ and within‐individual sources of variation. Second, defined in this way, repeatability tells us nothing about the mechanisms underpinning among‐individual differences. The latter point is important because there is a growing tendency to view *R* as a measure of how important ‘intrinsic’ mechanisms of phenotypic determination are. Taking an explicitly evolutionary perspective, several recent publications have argued that environmental effects can ‘confound’ repeatability estimates (Zsebők et al. 2017) and give rise to ‘pseudo‐repeatability’ (Harrison et al. 2015; Niemela and Dingemanse 2017) or—in a specifically behavioural context–‘pseudo‐personality’ (Martin and Réale 2008; Westneat et al. 2011). For instance, Niemela and Dingemanse (2017) define ‘true’ biological repeatability as arising ‘solely due to the combined influences of genetic variation and irreversible plasticity.’ The assumed irreversibility or ‘permanence’ of these mechanisms is stressed in contrast to reversible effects that lead to within‐individual variation. Under this definition, it follows that among‐individual differences arising from, for instance, reversible plastic responses to local environments are cited as a source of upward bias in *R*. However, it is important to recognise here that there is no estimator bias here in the usual statistical sense. Rather the (perceived) problem is that the estimate of *R* will depend on sources of variance (within‐ and/or between‐individual) that the researcher would have preferred to control for. With this recognised, then it is sensible to consider how statistical and/or experimental controls can best be implemented to condition estimates of *R* on these ‘nuisance’ sources of variance.

The narrower interpretation of repeatability described above may help us to focus attention on a subset of biological mechanisms of interest. It also draws attention to the useful ways in which experiments and/or analyses can best be designed to test biological hypotheses and make at least preliminary evolutionary inferences (e.g., a trait that is not repeatable will not be heritable). However, I suggest some caution is also warranted. Firstly, while controlling for specific nuisance variables is often sensible, any inference of mechanism underpinning simple variance partitions (e.g., V_I_) will remain strongly assumption‐laden. In other words, correlations cannot prove causation just because they are intra‐class correlations. Stronger inferences can of course be made when specific processes contributing to V_I_ are directly modelled (e.g., additive genetic inheritance, maternal effects, spatial autocorrelation). This point about V_I_ actually applies equally to the residual variance (V_R_) that can be interpreted, with caveats, as within‐individual variation arising from reversible plasticity. The caveats arise because (i) measurement error will also contribute to V_R_ and (ii) residuals are joint properties of observations recorded and the preferred statistical model as chosen by the researcher (see Brommer 2013 for more discussion of this). Secondly, this view risks divorcing *R* from its much more widely understood dependence on the full variance of observed data. In so doing it increases the likelihood of misunderstanding. If the goal is not to estimate R in the sample obtained, but rather to estimate R in an idealised population in which, for instance, environmental effects are experienced identically by all individuals (and do not contribute to V_I_) then this is legitimate. However, this intent and its rationale must be made explicit. If this is not done then claims of environmentally induced bias are at best confusing and at worst misguided.

For instance, imagine a resource‐dependent (i.e., plastic) trait in a population where animals show limited dispersal and inhabit a spatially patchy environment. If all animals are repeatedly assayed then habitat patchiness will contribute to among‐individual variance in the observed data. Conventionally, this environmental heterogeneity would thus be seen as one mechanism contributing to V_I_ and thus R. However, controlling for patch differences (statistically or experimentally) could equally be viewed as (i) removing upward bias (following a mechanistic definition of R (Niemela and Dingemanse 2017)), (ii) inducing downward bias by removing a source of among‐individual variance that is highly relevant to the real‐world population (and could potentially even be ‘intrinsic,’ for example in a territorial species with competitive outcomes determined by heritable traits), or (iii) giving rise to a different estimate of *R* that is equally valid, but should be interpreted as representing (expected) repeatability in a homogeneous environment. I tend towards the third option, but note that claims of bias (in either direction) are not incorrect provided they are appropriately contextualised.

## Conditioning Estimates of R on ‘Nuisance’ Variables

More generally it is widely argued that an individual's phenotype will depend on both intrinsic and extrinsic factors ‐ concepts that are convenient but may be difficult to cleanly separate in reality. However, it is not the case that intrinsic factors exclusively drive among‐individual variance while extrinsic ones lead to within‐individual variance. Indeed,some factors will certainly contribute to both among‐ and within‐individual partitions of observed variance, meaning they may either increase or decrease *R*. Age effects might provide one obvious example in a longer term study where individuals are observed across several years, but average observation age also differs among individuals. The conceptual separation of within‐ and among‐individual variance becomes even less clear cut if individuals differ in their plastic responses to some environmental variable (I × E), as V_I_ then becomes a function of E (Nussey et al. 2007).

Nonetheless, in the absence of experimental controls (which are of course a good thing; Niemela and Dingemanse 2017), it is possible and often highly desirable to estimate conditional or adjusted repeatabilities. These are estimates that control statistically for nuisance sources of variance (as deemed by the modeller) in a data set. At a minimum these might include experimental design variables such as time of day, test sequence or observer identity. Using linear mixed effect models this can be done in several ways (see Nakagawa and Schielzeth 2010; Killen et al. 2016 and Wilson 2008 for parallel points made with respect to heritability); fixed effects can be included to yield estimates of adjusted repeatability; random effects can be added to make further partitions of the variance that are simply excluded from the summation to V_P_ (an approach sometimes used for calculating heritabilities of personality traits; Dochterman et al. 2015); or bivariate models can be used to estimate repeatabilities of one trait conditional on a second (following methods analogous to those used to obtain conditional genetic parameters in quantitative genetic studies; Hansen and Houle 2008). Conversely, recent work by de Villemereuil et al. (2018) actually presents methods that allow the variance explained by some or all fixed effects to be deliberately retained in the estimation of V_P_. This flexibility is welcome as it allows researchers to decide what constitutes the ‘natural phenotypic variance of the studied population’ (de Villemereuil et al. 2018). However, this further highlights the point that that there is no single correct denominator for estimating R.

## Re‐scaling Traits to Estimate *R* can Sometimes be Very Misleading

Given the ready availability of the mixed models strategies noted above, the once common practice of estimating repeatability in ‘corrected data’ (i.e., residuals from a previous analysis) is no longer widespread. Nor indeed is it statistically justifiable. However, one further strategy, which appears at least initially attractive if trying to control for allometric relationships, is to re‐scale a trait of interest (Y) by some second attribute (X) of the individual – often a measure of body size. This is most commonly done for metabolic and endocrine traits where, for instance, whole‐organism measures of O_2_ consumption might be turned into mass‐specific metabolic rate. Among‐individual differences in physiology are central to several heuristic explanations for the maintenance of multivariate life history variation (e.g., pace of life syndrome, stress coping style). However, in testing these ideas evolutionary biologists must not uncritically adopt data scaling practices common in physiological studies. Wider criticism of the use of ratios is presented elsewhere (e.g., Hayes 2001), but in the present context the key point is that the variance structure of Y/X necessarily depends on the distributions of both constituent variables. This means that repeatable variation in X (e.g., body size) can be a sufficient condition to generate variation in Y/X.

**Figure 1:**
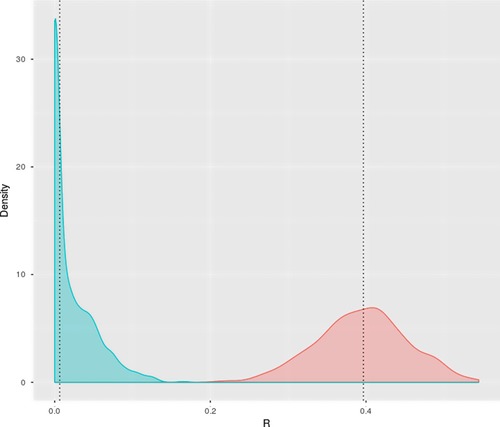
Distributions of estimated repeatability for Y following two possible “corrections” for X (mass) from simulated data sets (*n* = 1000). Plots show distributions under Model A in which the response variable was Y/X (red), and Model B in which the response was Y and X was included as a fixed effect. Dashed vertical lines indicate median estimates of *R*.

A toy simulation with arbitrary numbers serves to illustrate the potential for biological misinterpretation. Let body mass (X) be distributed in a population as X∼N(10,1), with an individual repeatability of R_X_ = 0.9. This might correspond to a scenario in which true mass varies among‐individuals, is constant within‐individuals, and is observed with (random) measurement errors ε_x_ where ε_x_ ∼N(0,0.1). Now let a trait Y have a simple linear allometry to (true) mass, assuming a slope of 1 and an intercept of 10. There is no among‐individual variance in Y over and above that caused by mass, but observations of Y are subject to random measurement errors ε_Y_, where ε_Y_ ∼N(0,1). A thousand data sets, in which 100 individuals were observed four times each, were simulated using these starting parameters and for each the scaled response Y/X was analysed using a linear mixed model (A) containing a fixed mean and a random effect of individual. For each simulated data set V_I_ was tested by likelihood ratio test (LRT, assuming the test statistic is distributed as χ^2^_1_) and an estimate of R obtained. For comparison, a second model (B) was also fitted to each data set, in which the unscaled trait Y was modelled with a (linear) fixed effect of X in addition to the mean and a random individual term. The distributions of repeatability estimates under the two models are shown in Figure 1. In model A, the median (95% quantiles) estimate of R for Y/X is 0.397 (0.285‐0.508) and the LRT is significant at α = 0.05 in 100% of cases. Under model B, the median estimate of adjusted R for Y is 0.007 (0.000‐0.096) and the LRT is significant in just 2.7% of cases. In neither case is the repeatability estimator biased in a statistical sense. However, if the biological question is whether there is more among‐individual variation in Y than can be explained by size differences alone, then use of Y/X as a response variable leads, in this instance, to entirely the wrong conclusion.

## Summary

In conclusion, there is not a single ‘true’ estimate of *R* for a trait in a population, but rather a set of equally valid estimates, each of which can be justified provided attention is paid to their subtle differences of biological interpretation. In some cases there may be a need to think carefully about whether particular scaling or transformations of a response variable could give misleading answers, but more generally differences will stem from the extent to which statistical controls are used to remove specific sources of variation in the trait of interest. They will also stem of course from experimental design decisions, including the use (or not) of experimental manipulations and sampling strategy (e.g., the inter‐observation interval). Decisions made will thus reflect the intent to exclude (or not) particular sources of within‐ and/or among‐individual variation. In evolutionarily motivated studies, this in turn may stem from a desire to interpret R in a narrower, more mechanistic way than has traditionally been the case, removing ‘extrinsic’ sources of among‐individual variance that are unlikely to impact evolutionary dynamics. There is ample room for plurality of practice and consequently I make no suggestions of how researchers should proceed, except to stress the need for clarity of intent (i.e. what do I intend my estimate of *R* to mean) and contextualisation (i.e., why is this the appropriate estimate for my study). A repeatability can mean different things to different people, and where one person may see a biological signal, another will see bias. A priori dismissing one of these perspectives provides obvious potential for confusion and fruitless debate and is hence unlikely to advance our understanding of both the causes and the consequences of individual variation.

Associate Editor: A. Charmantier

## Supporting information

Supplemental material
