# Supplementary material for: How should we interpret estimates of individual repeatability?
Source: Evol Lett. 2018 Jan 31;2(1):4–8. doi: 10.1002/evl3.40 (PMC6121803; doi:10.1002/evl3.40)
Supplement: Supplementary file 1 — Supplemental material [file EVL3-2-4-s001.docx]

**Wilson - How should we interpret estimates of individual repeatability?**

**Supplemental material: R code used for toy simulation**

library(lme4)

library(tidyverse)

library(broom)

Vi <- 0.90 #set Vi for mass

Vr <-(1-Vi) #set Vr for mass, assume this is measurement error

n<-100 # n=indiv

o<-4 # o=observations per ind

i<-1000 # i= number simulated data sets

resultsA<-matrix(0, 1000, 2) #create structures to store results

resultsB<-matrix(0, 1000, 2)

for (i in 1:i) { # i=number of data sets

IND<-as.vector(rep(1:n, o)) #list of ID's

id<-rnorm(n, mean=0, sd=(Vi^0.5)) #vector of individual effects

id2<-rep(id,o)

res<-rnorm((n*o), mean=0, sd=(Vr^0.5))

mass<-10+id2+res #create a mass variable, mean of 10

# Create a variable (e.g. metabolic rate, mr) with allometric slope to (true) mass of 1

# and an intercept of 10. Add some residual variance around that for the observations

# There is no repeatable variation added to MR beyond that coming from allometry to mass

res2<-rnorm((n*o), mean=0, sd=1^0.5) # vector of residuals, assume ~N(0,1)

mr<-10+1*(10+id2)+res2

#build the data file for each simulation iteration

simdat<-cbind(IND, id2, res, mass, res2, mr)

simdat<-as.data.frame(simdat)

colnames(simdat)<-c("IND", "id2", "res", "mass", "res2", "mr")

simdat$IND<-as.factor(simdat$IND) #specify ID as a factor for mixed model

simdat$mr_scaled<-simdat$mr/simdat$mass #create a Y/X variable, mr/mass

## FIT MODELS MIXED MODELS A & B PLUS REDUCED VERSIONS WITH NO IND EFFECT #############

#A) mr/mass

modelA1<-lmer(mr_scaled~1 + (1|IND), data=simdat)

modelA2<-lm(mr_scaled~1, data=simdat)

VInd_A<-tidy(modelA1, effects = "ran_pars", scales = "vcov")$estimate[1] # extract Vi

VRes_A<-tidy(modelA1, effects = "ran_pars", scales = "vcov")$estimate[2] # extract Vres

R_A<-VInd_A/(VInd_A+VRes_A) # calculate R of ratio

LRT_A1_A2 <- anova(modelA1, modelA2) # Perform LRT, refitting w/ ML

chi2_A<- LRT_A1_A2$Chisq[2]

P_A<- LRT_A1_A2$`Pr(>Chisq)`[2] #do LRT on VI on 1DF

resultsA[i,]<-cbind(R_A,P_A) #store repeatability and P value from LRT

#B) mr with mass as covariate

modelB1<-lmer(mr~1+mass + (1|IND), data=simdat)

modelB2<-lm(mr~1+mass, data=simdat)

VInd_B<-tidy(modelB1, effects = "ran_pars", scales = "vcov")$estimate[1] # extract Vi

VRes_B<-tidy(modelB1, effects = "ran_pars", scales = "vcov")$estimate[2] # extract Vres

R_B<-VInd_B/(VInd_B+VRes_B) # calculate R of mr conditional on mass

LRT_B1_B2 <- anova(modelB1, modelB2) # Perform LRT, refitting w/ ML

chi2_B<- LRT_B1_B2$Chisq[2]

P_B<- LRT_B1_B2$`Pr(>Chisq)`[2] #do LRT on VI on 1DF

resultsB[i,]<-cbind(R_B,P_B)

print(i) #print progress just to see how far along we are!

}

######COMPILE AND PLOT RESULTS#####################

results<-as.data.frame(cbind(resultsA,resultsB))

colnames(results)<-c("R_A", "P_A","R_B","P_B")

POWA<-length(results$P_A[results$P_A<=0.05])/length(results$P_A) # proportion sig

POWB<-length(results$P_B[results$P_B<=0.05])/length(results$P_B) # proportion sig

#summarise results for Model A

POWA

median(results$R_A)

quantile(results$R_A,c(0.025, 0.975)) #get 95 percentiles

#summarise results for Model B

POWB

median(results$R_B)

quantile(results$R_B,c(0.025, 0.975)) #get 95 percentiles

#Stack R estimates for easier plotting, then plot R distributions with medians

res2<-results[,c(1,3)]

res2<-stack(res2)

ggplot(res2, aes(x=values)) + geom_density(aes(group=ind, colour=ind, fill=ind), alpha=0.3) +

labs(x="R", y="Density") + theme(legend.position="none")+

geom_vline(xintercept=c(median(results$R_A), median(results$R_B)), linetype="dotted")
